# Genome and metabolome: chance and necessity

**DOI:** 10.1186/s13059-021-02501-0

**Published:** 2021-09-23

**Authors:** Emanuel Gonçalves, Christian Frezza

**Affiliations:** 1 Instituto Superior Técnico (IST), Universidade de Lisboa, 1049-001 Lisboa, Portugal; 2 INESC-ID, 1000-029 Lisboa, Portugal; 3 MRC (Medical Research Council) Cancer Unit, University of Cambridge, Cambridge, CB2 0XZ UK

The sequencing of large cohorts of cancer cell genomes provided an unprecedented depth to the characterization of somatic alterations associated with carcinogenesis and revealed a daunting complexity and heterogeneity, even within a single tumour. The interpretation of cancer driver alterations was further complicated as many of these mutations were also found in healthy cells. This apparent contradictory evidence supports the existence of evolutionary pressures that drive the selection of particular clones that confer a specific advantage in that context. Thus, in contrast to a simple accumulation or combination of key “driver” mutations, oncogenesis might be driven by the interplay between the availability of healthy cells harbouring oncogenic mutations along with permissive selective pressures. Environmental and nutrient cues can act as powerful “selectors” for specific genotypes. However, how they contribute to shaping clone dynamics is still unclear. Harnessing novel single-cell technologies and *in silico* metabolic models will enable the characterization of genomic, transcriptomic, proteomic, and metabolic heterogeneity during cancer development and reveal crucial insights into the processes driving clonal selections. Ultimately, this multidisciplinary approach will open novel avenues for therapies that could prevent or revert cancerous states and eradicate residual tumour cells to prevent cancer recurrence.

What drives healthy cells to acquire and sustain cancer phenotypes? Despite more than 50 years since discovering the first tumour suppressors and oncogenes, this fundamental question remains unaddressed. At every biological layer investigated so far, cancer cells exhibit dysregulated behaviour. Indeed, cancer cells show a myriad of genetic, epigenetic, transcriptomic, proteomic, and metabolic deregulations allowing them to, for example, sustain abnormal growth and evade the immune system [1]. Emerging data also indicate that these molecular and phenotypic alterations are highly heterogeneous across different patients or cancers, even within the same tumour.

With the advent of the human genome project and technological breakthroughs, cancer genomes are now routinely sequenced, enabling the systematic characterization of somatic genetic alterations observed in thousands of cancers. On average, each cancer contains 4 to 5 driver mutations, followed by a long tail of less frequently altered genes whose contribution to the disease is difficult to discern. For a small subset of cases, no driver mutations were detected [2]. Recent efforts to investigate the role of mtDNA mutations further showed that even the mitochondrial genome is highly mutated in cancer [3]. Yet, the interpretation of “driver” mutations in carcinogenesis is being reconsidered as recent studies started to probe the landscape of genetic alterations in healthy tissues and found that many of these driver events are also present in healthy cells [4, 5]. Taken together, these recent results underscored the importance of expanding our knowledge of the mutational processes in healthy cells and the early stages of neoplasm formation and carcinogenesis. Furthermore, these results questioned whether canonical driver mutations are truly driving tumour development, or in contrast, they reflect external environmental pressures that induce clonal selection where those mutations provide a fitness advantage.

**Fig. 1:**
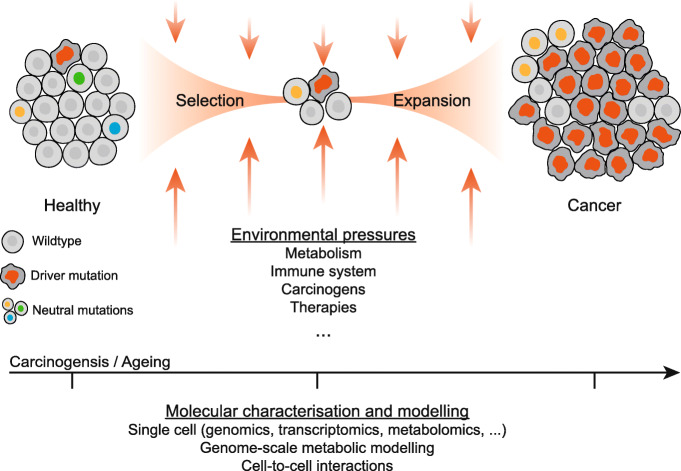
Schematic of the role of environmental selective pressures on genetically heterogeneous cell population leading to clonal selection and expansion of cells harbouring canonical cancer “drive” mutations. In the bottom, promising experimental and computational techniques are listed that together can be used to molecularly characterize the cellular changes throughout carcinogenesis, including modelling metabolism at single-cell resolution to discern the metabolic environmental pressures that drive clonal selection. Driver mutations, depicted by a red nucleus, represent cells harbouring genetic alterations that predispose to cancer phenotypes. Neutral mutations, shown by different coloured nuclei, showcase cells with genetic alterations that are not associated with cancer and are generally lost upon environmental selection

A long-standing hypothesis for tumour formation is that mutations observed are just the echo of a selection process shaped by metabolic and immune-cell driven cues within the tumour microenvironment [6]. Within this alternative model for carcinogenesis, selective pressures are the driver events of carcinogenesis by positively selecting a mutant cell already present in the cellular population (Fig. 1). These specific mutations confer a growth or a survival advantage over other healthy cells, making the mutant cell a precursor for a dominant cellular population. Of note, different mutations may converge to a similar molecular phenotype, enabling the coexistence of diverse and apparently disconnected “driver” mutations within a tumour. In line with this hypothesis, a recent study could not identify any discernible mutational signature induced by most 20 carcinogens tested in chronically exposed mice, and the driver mutations found could be attributed to endogenous cellular processes [7]. Furthermore, mounting evidence is becoming available of the role of non-genetic processes in cancer, in particular in driving resistance to therapies. A large study of metastatic cancers revealed almost perfect concordance between the genetic profiles of the first and second biopsy ~ 6 months apart from patients under standard-of-care treatments [8]. Another recent study identified, at single-cell resolution, cellular lineages that persist after therapies by undergoing a metabolic shift where overexpression of glutathione metabolism and antioxidant signatures is associated with proliferative capacity [9]. Lastly, genome-wide CRISPR-Cas9 screens unveiled the importance of the metabolic milieu in determining genetic dependencies and the same cell line in different metabolic contexts displays > 5% significant differential gene dependencies [10]. Altogether, these multiple lines of evidence suggest that non-genetic processes are important in cancer, and metabolism is an essential driving force in tumour development and resistance to therapies.

The role of metabolism in cancer is well established, and it supports and regulates cancer phenotypes, such as abnormal energy requirements and unrestrained proliferation [11]. Cancer metabolism, in contrast to genomics, is considered closer to the cellular phenotype and provides a more functional understanding of the cellular states and transitions. Metabolism is a dynamic process, characterized by constant catalysis of metabolic reactions, where both metabolite abundance, intracellular and extracellular, and reaction rates are essential to define a metabolic state. Importantly, metabolic fluxes cannot be easily inferred from transcriptomics analyses, highlighting a knowledge gap between genome analyses and the emerging phenotype [12]. These intrinsic features make metabolism complex to represent and technically challenging to measure, hindering their efficient application on a scale similar to that of genomic studies. Nonetheless, metabolomics is joining genomics, transcriptomics and proteomics and entering the single-cell revolution with recent technology developments which identified cellular populations with distinct metabolic profiles [13]. These approaches hold great promise to shed light on the diversity of metabolic profiles of cancer cells at different stages of carcinogenesis and reveal the selective pressures metabolism undergoes during clonal evolution.

The combinatorial space of molecular interactions and the contexts in which they can happen is mind-boggling, and while biology is not random, i.e. not everything can interact with everything, it is intractable to experimentally measure even a fraction of it. Computational models, even if not perfect, can help navigate this space and narrow down to more probable hypotheses at a scale that can be experimentally assessed, which in turn cycles back to further improve the model, completing the systems biology cycle. An example of this is genome-scale metabolic models, which provide an integrative framework to tailor metabolic networks to specific tissues or single cells using genomics, transcriptomics and proteomics datasets [14]. Moreover, the mathematical representation of the metabolic network enables scalable simulations of the metabolic fluxes and to predict the impact of genetic metabolic perturbations as well as alterations in metabolic environmental conditions. The continued technological developments, for example, multi-omics single-cell measurements, cellular imaging and base-pair resolved functional genetic screens, will enable the generation of comprehensive and complex molecular and phenotypic datasets. In particular, single-cell transcriptomics combination with genome-scale metabolic modelling revealed the diversity of T helper 17 (Th17) cells metabolic states and the role of specific metabolic pathways in Th17 function [15].

Looking ahead, studies spanning both cancer genomics and cancer metabolism will be in a privileged position to bridge knowledge of the genetic alterations that predispose to cancer with a functional understanding of the metabolic rewiring that enables and maintains cancer phenotypes. Focus into the early stages of neoplasm formation and to how healthy cells age and accumulate mutations will be important to discern which genetic alterations and metabolic states act as precursors for clonal expansions of cancerous cells. For decades we have been working on the footprint of cancer evolution (the genetic alteration), without fully understanding the evolutionary principles behind their selection. Mutations may arise by chance, but it is, at least in part, the metabolic fitness that they bring about that makes them necessary to face evolutionary pressure. Comprehensive studies to systematically characterize the selective pressures involved throughout carcinogenesis will allow the development of strategies to alter these pressures through changes to the tumour environment and ultimately impair cancer cell growth or even revert to a healthy or more therapeutically manageable state.
